# Effect of atrial fibrillation on outcomes after mechanical thrombectomy and long-term ischemic recurrence in patients with acute basilar artery occlusion

**DOI:** 10.3389/fneur.2022.909677

**Published:** 2022-07-29

**Authors:** Chenhao Zhao, Weidong Luo, Xing Liu, Jun Luo, Jiaxing Song, Junjie Yuan, Shuai Liu, Jiacheng Huang, Weilin Kong, Jinrong Hu, Jie Yang, Ruidi Sun, Chengsong Yue, Dongjing Xie, Linyu Li, Hongfei Sang, Zhongming Qiu, Fengli Li, Deping Wu, Wenjie Zi, Qingwu Yang

**Affiliations:** ^1^Department of Neurology, Xinqiao Hospital and The Second Affiliated Hospital, Army Medical University (Third Military Medical University), Chongqing, China; ^2^Department of Medicine, Xinqiao Hospital and The Second Affiliated Hospital, Army Medical University (Third Military Medical University), Chongqing, China; ^3^Department of Neurology, The 404th Hospital of Mianyang, Mianyang, China; ^4^Huaian Medical District of Jingling Hospital, Medical School of Nanjing University, Huaian, China

**Keywords:** acute basilar artery occlusion, atrial fibrillation, mechanical thrombectomy, ischemic recurrence, recurrence

## Abstract

**Introduction:**

According to the literature on anterior circulation, comorbid atrial fibrillation (AF) is not associated with a worse functional outcome, lower reperfusion rates, or higher rates of intracranial hemorrhage after mechanical thrombectomy (MT) compared to intravenous thrombolysis (IVT) or treatment with supportive care. However, data are limited for the effect of comorbid AF on procedural and clinical outcomes of acute basilar artery occlusion (ABAO) after MT. This study aimed to investigate the effect of atrial fibrillation on outcomes after MT and long-term ischemic recurrence in patients with ABAO.

**Methods:**

We performed a registered study of the Endovascular Treatment for Acute Basilar Artery Occlusion Study (BASILAR, which is registered in the Chinese Clinical Trial Registry, http://www.chictr.org.cn; ChiCTR1800014759) from January 2014 to May 2019, which included 647 patients who underwent MT for ABAO, 136 of whom had comorbid AF. Prospectively defined baseline characteristics, procedural outcomes, and clinical outcomes were reported and compared.

**Results:**

On multivariate analysis, AF predicted a shorter puncture-to-recanalization time, higher first-pass effect rate, and lower incidence of angioplasty and/or stenting (*p* < 0.01). AF had no effect on intracranial hemorrhage incidence [adjusted odds ratio (aOR), 1.093; 95% confidence interval (CI), 0.451–2.652], 90-day functional outcomes (adjusted common odds ratio, 0.915; 95% CI, 0.588–1.424), or mortality (aOR, 0.851; 95% CI, 0.491–1.475) after MT. The main findings were robust in the subgroup and 1-year follow-up analyses. Comorbid AF was the remaining predictor of ischemic recurrence (aOR, 4.076; 95% CI, 1.137–14.612).

**Conclusions:**

The study revealed no significant difference in the safety and efficacy of MT for ABAO regardless of whether patients had comorbid AF. However, a higher proportion of patients with AF experienced ischemic recurrence within 1 year after MT.

## Introduction

Stroke remained the second leading cause of death worldwide in 2019 and is associated with the highest disability-adjusted life years lost to any disease in China (1, 2). Atrial fibrillation (AF) is an important contributor to ischemic stroke. The proportion of cardioembolic stroke in China (about 10%) remains lower than in high-income countries (about 30%) (3), which is apparently due to the underdiagnosis of AF. Considering the aging population and the high proportion of undertreated patients in clinical practice, AF has remained an important and common risk factor for acute ischemic stroke for a long time (4).

Large registry studies (Canada, 2013; Japan, 2005; Austria, 2004) and a retrospective study (America, 2011) have demonstrated that comorbid AF is an independent predictor of poor functional outcomes and increased mortality after an ischemic stroke after intravenous thrombolysis (IVT) (5–8), and one study (Turkey, 2016) has demonstrated that AF-associated acute ischemic stroke is related to a higher risk of unfavorable functional outcomes and a higher proportion of complications after mechanical thrombectomy (MT) (9). However, a recent retrospective (America, 2021) analysis showed that MT influences the effects of AF in ischemic stroke (10). Remarkably, these data were mainly from patients with either an anterior circulation stroke or unselected stroke, and studies of posterior circulation stroke have not been reported.

We speculate whether comorbid AF would have different effects on patients with acute basilar artery occlusion (ABAO) after MT, based on factors such as blood supply territory, heterogeneity of ischemic tolerance, and high variation in clinical manifestation as well as the presence of potentially rich collateral circulation, which might exert an impact on the neurological outcome (11, 12).

Additionally, AF remains a common high-risk condition for recurrent ischemic stroke. Anticoagulation is generally recommended in patients with AF and stroke or transient ischemic attack. Perioperative antithrombotic therapies are also associated with the risk of intracranial hemorrhage and recurrent ischemic events (13). Although we have observed this phenomenon in long-term follow-ups of patients, studies on real-world data emphasizing the characteristics, clinical outcomes, and ischemic recurrence of ABAO with AF are rare.

We, therefore, aimed to identify the relevant treatment profiles of MT in the Endovascular Treatment for Acute Basilar Artery Occlusion Study (BASILAR) registry; demonstrate differences in procedural efficiency, functional outcomes, and complications in patients who had AF and underwent MT for ABAO; and explore the potential risk factors for long-term ischemic recurrence.

## Materials and methods

### Study design and population

Patient data were drawn from the BASILAR registry, which was registered with the Chinese Clinical Trial Registry (http://www.chictr.org.cn; ChiCTR1800014759). Briefly, this nationwide, multicenter, prospective, investigator-initiated registry study was designed to investigate the efficacy and safety of endovascular treatment in patients with ABAO. For the present analysis, among 829 patients in the full registry cohort, we included 647 patients who underwent MT for ABAO at 45 comprehensive stroke sites between January 2014 and May 2019. Among the 647 patients, 136 had AF and 511 did not have AF. Only patients with available information on AF status before the stroke episode or during the hospital stay were included and followed up for 1 year. Further details of the BASILAR registry have been published previously (14).

Acute basilar artery occlusion was confirmed using computed tomographic angiography, magnetic resonance angiography, or digital subtraction angiography within 24 h of the estimated occlusion time. AF was diagnosed at the discretion of appropriately trained personnel at each site, usually based on the detection of different findings from routine electrocardiogram monitoring and 24-h Holter recording, per the current standard practice (15). Patients were, regardless of AF pattern or burden, divided into the AF group if they had a known or new diagnosis of AF or the non-AF group (16). Neurological deficit was quantified using the National Institutes of Health Stroke Scale (NIHSS) to assess stroke functional severity (17). Ischemic changes were quantified using the posterior circulation Alberta Stroke Program Early Computed Tomography Score (pc-ASPECTS, range 0–10, with scores of ≥8 being correlated with a favorable outcome) (18). The presumed stroke causative mechanism was assessed based on the Trial of ORG 10,172 in Acute Stroke Treatment (TOAST) classification (19).

### Mechanical thrombectomy

Patient selection for MT was left to the discretion of each operator or his/her consultation and discussion with patient representatives, and this was performed independent of the present study. The frontline thrombectomy approach used was based on the operator's preference and included a stent retriever or, in a few cases, thromboaspiration, balloon angioplasty, stenting, or a combination of these approaches. The procedural outcome of MT was assessed using the modified thrombolysis in cerebral infarction (mTICI) scale (20). Successful recanalization was defined as an mTICI score of 2b or 3 at the end of the procedure, as confirmed by imaging core laboratory results according to individual angiography data. The first-pass effect (FPE), defined as achieving complete recanalization after a single thrombectomy device, was used without rescue therapy (21). Procedural notes were reviewed for technological complications, such as the type of arterial perforation, dissection, embolization in a new territory, vasospasm, and vascular rupture during the interventional procedures.

### Follow-up and outcome measures

Clinical outcomes were assessed using modified Rankin Scale (mRS) scores by trained stroke neurologists at each site, during an outpatient visit, at 90 days (± 2 weeks), and at 12 months (± 4 weeks) after treatment either in the outpatient clinic or *via* telephone interviews if patients were unable to visit the outpatient clinic. Due to the incomplete follow-up data of stroke recurrence within 90 days, we obtained recurrence data from 90 days to 1 year.

Outcome measures at the 90-day follow-up were as follows: (1) the primary outcome was a shift in the mRS score [ordinal, adjusted common odds ratio (acOR), per point increase], which was estimated using ordinal logistic regression analysis (shift analysis). The mRS assesses the level of disability ranked between 0 and 6, with 0–3 indicating moderate functional outcome, 4–5 indicating an increased level of disability, and 6 indicating death (22). (2) Moderate functional outcome defined as an mRS score of 0–3 was also evaluated in the sensitivity analysis. (3) Symptomatic intracranial hemorrhage (sICH) within 48 h after MT was assessed using the Heidelberg Bleeding Classification (23). (4) Lastly, all-cause mortality was evaluated.

Outcome measures at the 1-year follow-up were as follows: (1) proportions of long-term moderate functional outcomes, (2) all-cause mortality, and (3) 1-year ischemic recurrence, defined as a composite of recurrent stroke, transient ischemic attack, and symptomatic systemic embolism. Although our definition of recurrent ischemic stroke was not identical to that in published literatures, it followed the definition used in cardiological practice, which corresponds to an acute focal neurologic deficit, presumably due to ischemia that either resulted in clinical symptoms lasting ≥ 24 h or was associated with evidence of relevant infarction on cerebral imaging (24).

### Statistical analysis

Univariate comparisons of prospectively defined baseline characteristics, treatment profiles, and clinical outcomes between patients presenting with and without AF were summarized using the Mann–Whitney *U* test for independent numerical variables (all of which followed a non-normal distribution) or ordinally scaled variables, and the Pearson χ^2^ test or Fisher exact test for categorical variables.

To investigate whether the AF status was an independent predictor of the treatment profiles, a logistic regression model was used to assess the categorical outcomes [e.g., FPE and use of percutaneous transluminal angioplasty and/or stenting (PTA/PTAS)], and a linear regression model was used to evaluate continuous outcomes (e.g., procedure time), with adjustment of the following confounders: age (continuous), sex (categorical), diabetes mellitus (DM, categorical), dyslipidemia (categorical), the admission NIHSS score (continuous), admission pc-ASPECTS (continuous), location of ABAO (categorical; contrast type: comparator; indicator: distal third segment), intravenous thrombolysis (IVT, categorical), and time from symptom onset to vessel puncture (OTP, continuous).

Predictors were identified using two models for clinical outcomes and ischemic recurrence: (1) for the interventional model, the association of comorbid AF with all of the previously listed outcome measures was assessed using multivariable ordinal and binary logistic regression analyses adjusted for the following confounders: age, DM, the admission NIHSS score, admission pc-ASPECTS, location of the occlusion, IVT, time from groin puncture to vessel recanalization (PTR, continuous), and an mTICI score of 0–2a vs. a TICI score of 2b−3 (categorical). (2) To address the issue of ischemic recurrence, we added potential risk factors such as age, systolic blood pressure (continuous), glycated hemoglobin A1c level (continuous), and cigarette smoking (categorical) to the risk factor model.

Subgroup analyses were also performed to investigate the consistency of the AF conclusions of the primary analysis among different subpopulations based on various dichotomizations of baseline characteristics of patients with MT. Given center-to-center variability in patient demographics that may have introduced bias into the comparison of outcomes between cohorts, we sought to determine whether this variability affected the conclusions by including the treating centers [categorical, contrast type: comparator; indicator: largest center (*n* = 69)] as a variable in the multivariate analysis.

All analyses were based on the intention-to-treat principle. The rationale for the aforementioned models was the combination of prespecified variables of outcome following MT and some baseline variables (*p* < 0.05) in univariate testing (14). The enter method of logistic regression analysis was used in the multivariate analysis. The rates of missingness for key baseline variables and outcomes in this study were low [e.g., admission pc-ASPECTS and anticoagulation, 4/647 (0.6%); PTR, 3/647 (0.5%); sICH, 11/647 (1.7%); loss to follow-up at 1-year, 32/647 (4.9%)]; missing values for select key variables were analyzed with complete cases. All statistical significance values were set at *p* < 0.05, and all *p*-values were two-sided. All statistical analyses were performed using SPSS version 26.0 (IBM Corp., Armonk, NY).

## Results

### Baseline characteristics

Of the 647 patients [74.7% male, median age 64 years (range, 56–73)], 136 patients who had comorbid AF were more likely to be older and female, had vascular and valvular heart disease, and lower rates of dyslipidemia (*p* < 0.05) than the 511 patients without AF. The data also showed that patients with comorbid AF had a higher incidence of a maximum neurological deficit from the onset and more severe symptoms on admission than the 511 patients without AF, and the occlusion tended to occur in the distal basilar artery. A non-significantly shorter OTP and a significantly shorter PTR were found in the AF cohort than in the non-AF cohort (shown in Table 1).

**Table 1:** Baseline characteristics and treatment profiles according to AF status.

**Outcome**	**Available of** ***n*** = **647**	**AF (*****n*** = **136)** **Median (IQR)/N (%)**	**No-AF (*****n*** = **511)** **Median (IQR)/N (%)**	***p*** **-value**
Baseline characteristics				
Age (years)	647	73 (65–78)	63 (55–70)	<0.001
Sex (female)	647	63 (46.3)	101 (19.8)	<0.001
Maximum deficit from onset	647	78 (57.4)	192 (37.6)	<0.001
Admission NIHSS	647	30 (22–34)	25 (16–32)	0.001
Admission pc–ASPECTS	643	8 (7–10)	8 (7–9)	0.082
Hypertension	647	92 (67.6)	359 (70.3)	0.556
DM	647	27 (19.9)	122 (23.9)	0.322
Dyslipidemia	452	33 (37.5)	189 (51.9)	0.015
Previous TIA/AIS	647	25 (18.4)	120 (23.5)	0.249
CHD	647	48 (35.3)	57 (11.2)	<0.001
VHD	647	16 (11.8)	2 (0.4)	<0.001
INR	557	1.06 (1.00–1.15)	1.02 (0.96–1.09)	<0.001
Medication history				
Antiplatelet	644	34 (25.0)	135 (26.6)	0.794
Anticoagulation	643	12 (9.0)	1 (0.2)	<0.001
Statin	644	20 (14.7)	74 (14.6)	0.967
Stroke causative mechanism	647			<0.001
LAA		3 (2.2)	415 (81.2)	
CE		130 (95.6)	43 (8.4)	
Others†		3 (2.2)	53 (10.4)	
Location of ABAO	647			<0.001
Distal third		104 (76.5)	118 (23.1)	
Middle third		17 (12.5)	178 (34.8)	
Proximal third		7 (5.1)	100 (19.6)	
VA–V4‡		8 (5.9)	115 (22.5)	
Treatment profiles				
IVT§	647	25 (18.4)	94 (18.4)	0.997
OTP (min)	644	315 (221–462)	329 (220–501)	0.277
PTR (min)	644	91 (60.5–128)	109 (75–155)	0.001
PTA/PTAS	647	16 (11.8)	289 (56.6)	<0.001
Type of mechanical thrombectomy				0.012
Stent retriever	482	113(23.4%)	369 (76.6%)	
Aspiration	20	4(20%)	16(80%)	
PTA/PTAS	66	4 (3.1%)	62 (96.9%)	
Intra–arterial medication and/or mechanical fragmentation	75	61(81.3%)	14(18.7%)	
Combination of mechanical thrombectomy	422	55(40.4%)	367(71.8%)	<0.001
mTICI ≥ 2b	647	111 (81.6)	411 (80.4)	0.85
First pass effect	291	36 (41.9)	51 (24.9)	0.004
Craniectomy/Craniopuncture	647	6 (4.4)	8 (1.6)	0.09
Technological complications	646	19 (14.0)	49 (9.6)	0.141
Complications		•2 Arterial Perforation •2 Dissection •8 Distal Embolization •5 Vasospasm •2 Vascular Rupture	•5 Arterial Perforation •8 Dissection •19 Distal Embolization •13 Vasospasm •4 Vascular Rupture	

*AF, atrial fibrillation; IOR, interquartile rage; N, number; NIHSS, National Institutes of Health Stroke Scale; pc-ASPECTS, posterior circulation-Alberta Stroke Program Early Computed Tomography Score; DM, Diabetes mellitus; TIA/AIS, transient ischemic attack/Acute ischemic stroke; CHD, coronary heart disease; VHD, valvular heart disease; INR, International Normalized Ratio; LAA, large artery atherosclerosis; CE, cardioembolism; ABAO, acute basilar artery occlusion; VA-V4, Vertebral artery-V4; IVT, intravenous thrombolysis; OTP, time from symptoms onset to vessel puncture; PTR, time from groin puncture to vessel recanalization; PTA/PTAS, percutaneous transluminal angioplasty and/or stenting; mTICI, modified Thrombolysis in Cerebral Infarction; FPE, first pass effect.*

*†The definition of the distal vertebral artery (VA-V4 segment) occlusion included in this study referred to V4 segment occlusion of isolated vertebral artery.*

*‡The definition of others included small-vessel occlusion,stroke of other determined etiology, and undetermined etiology.*

*§The definition of IVT is administration of intravenous alteplase within 4.5 h or intravenous urokinase within 6 h of the estimated time of ABAO*.

### Treatment profiles

Although there was a shorter OTP, shorter PTR, and lesser use of PTA/PTAS in the AF cohort than in the non-AF cohort, MT was similarly effective in both cohorts, with the achievement of an mTICI score of ≥ 2b in 81.6% and 80.4% of patients, respectively (*p* = 0.850). The proportion of FPE in the AF cohort was higher than that in the non-AF cohort (41.9% vs. 24.9%, *p* = 0.004) (shown in Table 1).

**Figure 1:**
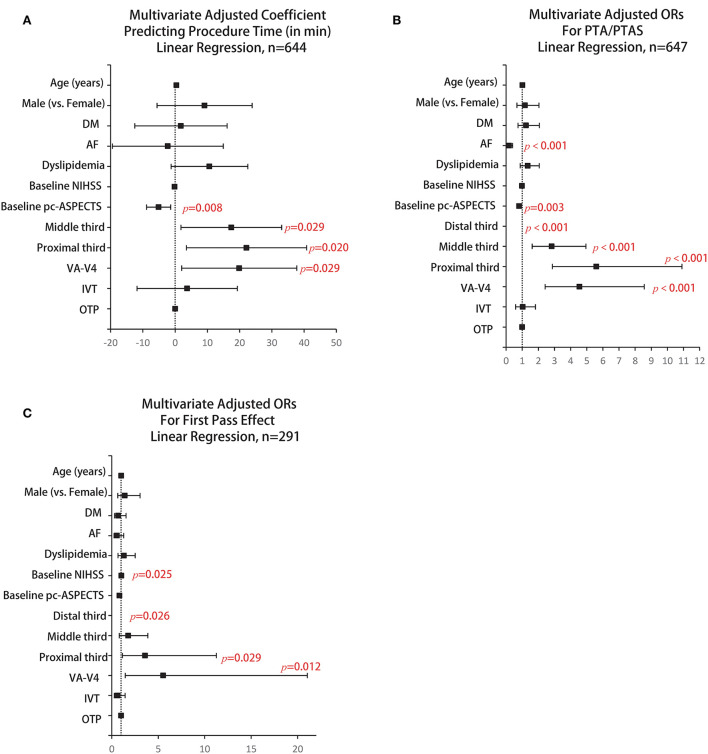
Results of multivariate regression analyses of predictors of procedure time. **(A)** maneuver-pass count, **(B)** and first-pass effect, **(C)** the coefficients and adjusted ORs and their estimates are shown, with error bars representing 95% CIs. Significant estimates (*p* < 0.05) are highlighted in red. AF, atrial fibrillation; DM, diabetes mellitus; NIHSS, National Institutes of Health Stroke Scale; pc-ASPECTS, posterior circulation Alberta Stroke Program Early Computed Tomography Score; VA-V4, vertebral artery-V4 segment; IVT, intravenous thrombolysis; OTP, time from symptom onset to vessel puncture; PTA/PTAS, percutaneous transluminal angioplasty and/or stenting; OR, odds ratio; CI, confidence interval.

To better determine whether AF status was an independent predictor of treatment profiles, we performed multivariate analyses adjusted for age, sex, DM, dyslipidemia, the admission NIHSS score, admission pc-ASPECTS, location of ABAO, IVT, and OTP. Multivariate linear regression analysis showed that comorbid AF was significantly associated with shorter PTR (adjusted coefficient, −2.257; 95% CI, −19.442 to 14.929; *p* = 0.796) (shown in Figure 1A). In addition, comorbid AF was significantly associated with a less use of PTA/PTAS [adjusted odds ratio (aOR), 0.192; 95% CI, 0.091–0.406; *p* < 0.001] (shown in Figure 1B) but not with FPE (aOR, 0.565; 95% CI, 0.249–1.284; *p* = 0.173) (shown in Figure 1C). Other predictors of treatment profiles are presented in Figure 1.

### Ninety-day follow-up outcomes

**Figure 2:**
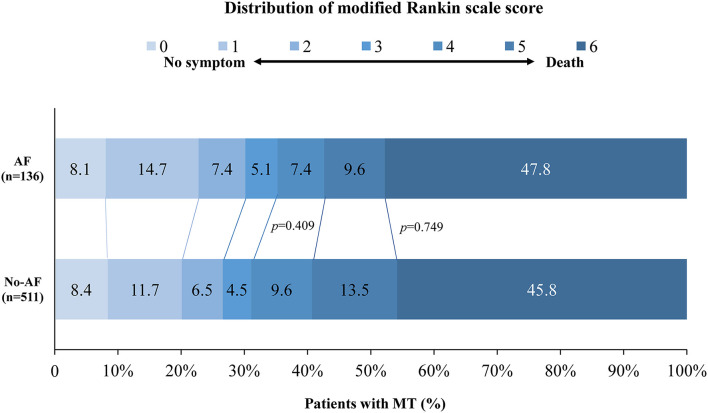
Distribution of modified Rankin scale scores at 90 days in patients with MT. The distribution shows that there was no statistically significant difference in moderate outcomes and mortality between the AF and non-AF cohorts. AF, atrial fibrillation; MT, mechanical thrombectomy.

All patients completed 90 days of follow-up. The univariate analysis showed that the AF cohort had similar efficacy outcomes to the non-AF cohort. The median [interquartile range (IQR)] values of the mRS score in the AF and non-AF cohorts were both 5 (2–6) (*p* = 0.902) in the univariate analysis (shown in Table 2). Likewise, the proportions of patients with moderate outcomes (35.3 and 31.1%, respectively; *p* = 0.409) and death (47.8 and 45.8%, respectively; *p* = 0.749) were comparable (shown in Figure 2). After adjustments were made in the interventional model, we observed an acOR for any improvement in the distribution of the mRS score (acOR, 0.915; 95% CI, 0.588–1.424, *p* = 0.694), favoring neither the AF nor the non-AF cohort. There were no significant differences in the moderate functional outcome (aOR, 1.093; 95% CI, 0.608–1.965, *p* = 0.765), mortality (aOR, 0.851; 95% CI, 0.491–1.475, *p* = 0.565), or sICH (aOR, 1.093; 95% CI, 0.451–2.652, *p* = 0.844) between the cohorts (shown in Table 2). After additionally adjusting the model for the treating centers, the main results showed no difference (acOR, 0.898; 95% CI, 0.563–1.433, *p* = 0.653) (reference: the largest center). Predictors associated with the improvement of the mRS score according to the shift analysis in patients with MT (*n* = 647) included age, DM, the admission NIHSS score, admission pc-ASPECTS, PTR, and an mTICI score of ≥ 2b, and age was no longer a predictor of functional outcome in the AF cohort (*n* = 136) (shown in Table 3).

**Table 2:** 90-day and 1-year follow-up outcomes of AF on univariate and multivariate analysis.

**Outcome**	**Available of** ***n*** = **647**	**AF (*****n*** = **136)** **Md (IQR)/N (%)**	**No-AF** **(*****n*** = **511)** **Md (IQR)/N (%)**	***p*** **-value**	**cOR (95% CI)**	***p*** **-value**	aOR*(95% CI)	***p*** **-value**
90-day follow-up								
mRS	647	5 (2–6)	5 (2–6)	0.902	0.978 (0.692–1.382)	0.899	0.915 (0.588–1.424)†	0.694
mRS 0-3	647	48 (35.3)	159 (31.1)	0.409	1.208 (0.811–1.799)	0.354	1.093 (0.608–1.965)	0.765
Mortality	647	65 (47.8)	234 (45.8)	0.749	1.084 (0.742–1.583)	0.677	0.851 (0.491–1.475)	0.565
sICH (Heidelberg definition)	636	11 (8.2)	34 (6.8)	0.699	1.231 (0.606–2.499)	0.565	1.093 (0.451–2.652)	0.844
1-year follow-up								
mRS 0-3	615	43 (32.3)	176 (36.5)	0.372	0.831 (0.552–1.249)	0.373	0.908 (0.504–1.636)	0.747
Mortality	615	79 (59.4)	257 (53.3)	0.213	1.281 (0.868–1.891)	0.213	1.216 (0.697–2.123)	0.491
Ischemic recurrence (beyond 90 days)	316‡	12 (17.6)	20 (8.1)	0.020	2.443 (1.128–5.292)	0.024	4.076 (1.137–14.612)§	0.031

*AF, atrial fibrillation; Md, median; IOR, interquartile rage; N, number; cOR, crude odds ratio; CI, confidence interval; aOR, adjusted odds ratio. mRS, modified Rankin Scale; sICH, Symptomatic intracranial hemorrhage.*

^*^Adjusted estimates of effect were calculated using multiple regression taking the following variables into account: age, DM, admission nihss, admission pc-ASPECTS, Location of ABAO, IVT, PTR, mTICI.

*†The adjusted common odds ratio was estimated from an ordinal logistic regression model and indicates the odds of improvement of 1 point on the mRS, with a common odds ratio greater than 1 indicating AF better.*

*‡The number of ischemic recurrences in AF and No-AF cohort was 68 and 248 respectively. Because of the incomplete follow-up data of ischemic recurrence within 90 days, there were only 316 cases remained after excluding 299 deaths within 90 days and 32 cases who were lost to follow-up at 1 year. The number of ischemic recurrences in AF and No-AF cohort was 68 and 248 respectively.*

*§Adjusting for AF, age, SBP, HbA1c, cigarette*.

**Table 3:** Multivariate analysis for predictors of improvement in 90-day mRS in the full cohort and in the AF cohort.

**Variable**	**Full cohort (*****n*** = **647)**				**AF cohort (*****n*** = **136)**			
	**ccOR (95%CI)**	***p*** **-value**	**acOR (95%CI)**	***p*** **value**	**ccOR (95%CI)**	***p*** **value**	**acOR (95%CI)**	***p*** **value**
age	1.019 (1.007–1.031)	0.002	1.019 (1.005–1.033)	0.007	1.04 (1.008–1.073)	0.013	1.003 (0.968–1.039)	0.864
DM	1.887 (1.329–2.68)	<0.001	1.783 (1.214–2.617)	0.003	5.123 (1.948–13.472)	0.001	5.909 (2.056–16.982)	0.001
AF	0.978 (0.692–1.382)	0.899	0.915 (0.588–1.424)	0.694	NA		NA	
Admission NIHSS	1.096 (1.079–1.114)	<0.001	1.095 (1.077–1.114)	<0.001	1.118 (1.078–1.16)	<0.001	1.102 (1.059–1.147)	<0.001
Admission pc-ASPECTS	0.642 (0.583–0.707)	<0.001	0.69 (0.622–0.765)	<0.001	0.597 (0.48–0.743)	<0.001	0.755 (0.591–0.965)	0.025
Location								
Distal third*	Ref		Ref		Ref		Ref	
Middle third	1.229 (0.865–1.746)	0.250	1.194 (0.784–1.819)	0.409	0.901 (0.352–2.308)	0.828	0.850 (0.295–2.450)	0.763
Proximal third	1.262 (0.827–1.927)	0.280	1.069 (0.654–1.747)	0.791	0.707 (0.178–2.815)	0.623	0.283 (0.058–1.389)	0.120
VA-V4	1.291 (0.862–1.934)	0.216	1.223 (0.765–1.960)	0.402	0.721 (0.197–2.644)	0.622	0.852 (0.204–3.549)	0.826
IVT	0.946 (0.658–1.36)	0.763	0.916 (0.618–1.358)	0.663	1.451 (0.639–3.298)	0.374	1.768 (0.708–4.417)	0.223
PTR	1.006 (1.004–1.009)	<0.001	1.007 (1.004–1.01)	<0.001	1.015 (1.008–1.022)	<0.001	1.015 (1.006–1.023)	0.001
mTICI ≥2b	0.157 (0.099–0.25)	<0.001	0.174 (0.106–0.286)	<0.001	0.114 (0.036–0.358)	<0.001	0.252 (0.074–0.858)	0.027

*mRS, modified Rankin Scale; AF, atrial fibrillation; ccOR, crude common odds ratio; acOR, adjusted common odds ratio; DM, Diabetes mellitus; NIHSS, National Institutes of Health Stroke Scale; pc-ASPECTS, posterior circulation-Alberta Stroke Program Early Computed Tomography Score; VA-V4, Vertebral artery-V4; IVT, intravenous thrombolysis; PTR, time from groin puncture to vessel recanalization; mTICI, modified Thrombolysis in Cerebral Infarction.*

*Distal third of the basilar artery was taken as a reference.

**Figure 3:**
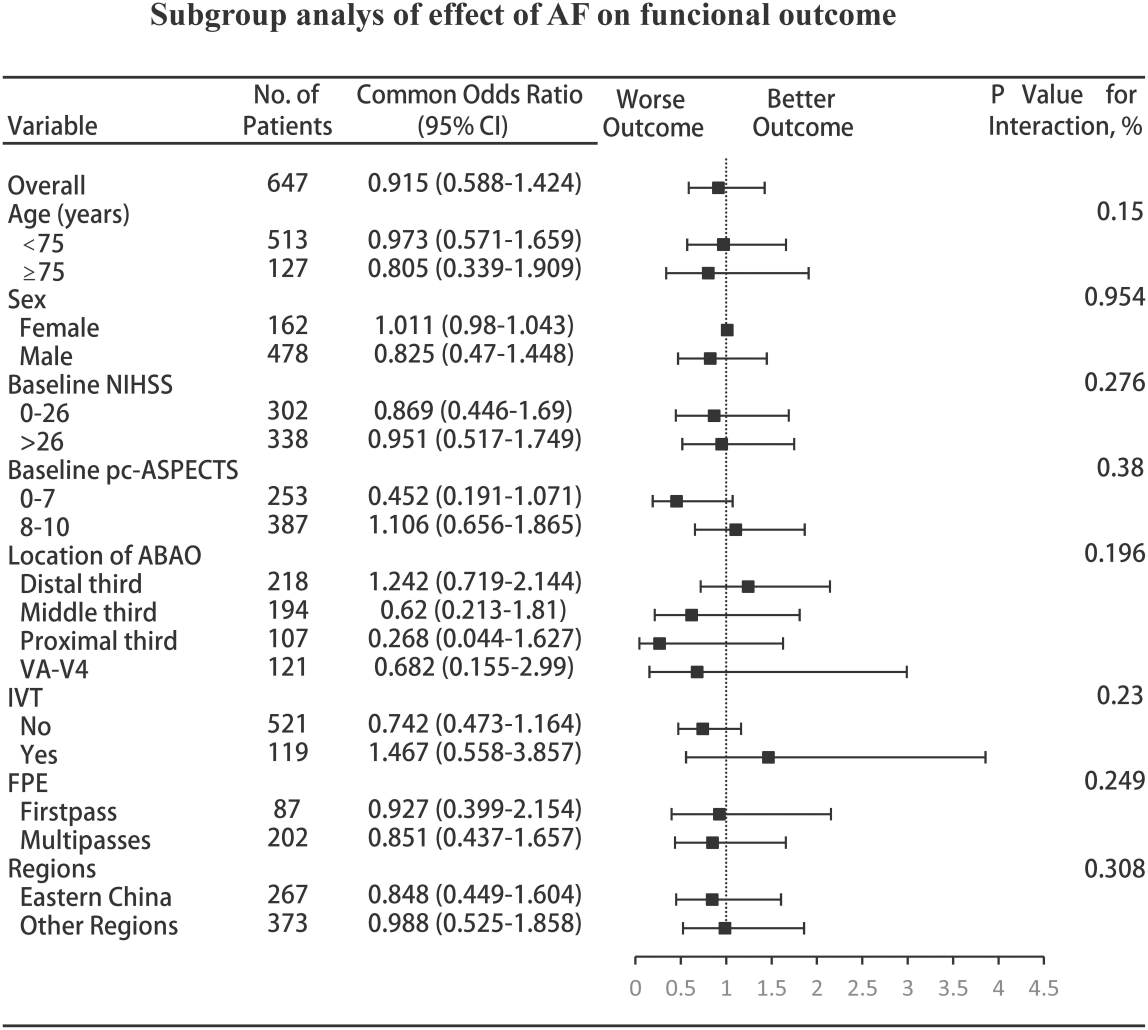
Subgroup analyses of the effect of AF on functional outcome. The forest plot shows that the differences in the improvement of 1 point on the mRS at 90 days, analyzed with the ordinal logistic regression analysis, favored neither patients with AF nor patients without AF across all prespecified subgroups; however, AF with moderate to severe ischemic change (admission pc-ASPECTS, 0–7) approached significance for increasing the odds of worse outcomes. The thresholds for the baseline NIHSS score and baseline pc-ASPECTS were chosen at the median, and the thresholds for age were chosen at the 75^th^ percentile. Regions were categorized into five regions: Eastern, Central, Southern, Southwestern, and Northeastern China. AF, atrial fibrillation; CI, confidence interval; NIHSS, National Institutes of Health Stroke Scale; pc-ASPECTS, posterior circulation Alberta Stroke Program Early Computed Tomography Score; ABAO, acute basilar artery occlusion; VA-V4, vertebral artery-V4 segment; IVT, intravenous thrombolysis; FPE, first-pass effect.

In almost all subgroups, including those based on age, sex, the admission NIHSS, admission pc-ASPECTS, location of ABAO, IVT, OTP, FPE, and geographic regions (to avoid bias of centric maldistribution: categorical, Eastern China [largest region] vs. other regions), subgroup analyses showed that no more information was extracted, but the remaining consistency of the primary analysis showed that comorbid AF did not affect the shift in the distribution of the mRS score; however, AF with moderate to severe ischemic change (admission pc-ASPECTS, −7) approached significance for increasing the odds of worse outcomes (acOR, 0.452; 95% CI, 0.191–1.071, *p* = 0.071) (shown in Figure 3).

### One-year follow-up outcomes

Of the 647 patients, 615 (95%) completed the 1-year follow-up visits and evaluations. No significant differences were found in moderate functional outcome (aOR, 0.908; 95% CI, 0.504–1.636, *p* = 0.747) or mortality between the AF and non-AF cohorts at 1 year (shown in Table 2). When adjusting for age, hypertension, DM, and cigarette smoking, we found that only ischemic recurrence was associated with long-term functional outcomes (aOR, 0.412; 95% CI, 0.193–0.876, *p* = 0.021).

During this follow-up period, only 316 cases remained after excluding 299 deaths within 90 days and 32 patients who were lost to follow-up at 1 year. Of these, 32 (10.1%) patients experienced ischemic recurrence, including 12 (17.6%) with AF and 20 (8.1%) without AF (shown in Table 2). Univariate comparisons of patients according to ischemic recurrence are presented in Table 4. The proportion of AF (37.5% vs. 19.7%, *p* = 0.024) and median age (72 vs. 63 years, *p* = 0.001) were significantly different between the AF and non-AF cohorts, and both comorbid AF and older age were associated with ischemic recurrence. However, after adjusting for baseline age and risk factors, we found that older age was not significantly associated with recurrence (aOR, 1.049; 95% CI, 0.991–1.111, *p* = 0.096). Among the risk factors, comorbid AF was the remaining predictor of ischemic recurrence (aOR, 4.076; 95% CI, 1.137–14.612, *p* = 0.031).

**Table 4:** Univariate and multivariate analysis of the demographics and risk factors in the cohort of ischemic recurrence (*n* = 316).

**Variables**	**Recurrence (*****n*** = **32) Md (IQR)/N (%)**	**No recurrence (*****n*** = **284)** **Md (IQR)/N (%)**	***p*** **-value**	**cOR (95% CI)**	***p*** **-value**	aOR*(95% CI)	***p*** **-value**
Age (years)	72 (60–79)	63 (54–71)	0.001	1.052 (1.015–1.089)	0.005	1.049 (0.991–1.111)	0.096
AF	12 (37.5)	56 (19.7)	0.020	2.443 (1.128–5.292)	0.024	4.076 (1.137–14.612)	0.031
SBP (mmHg)	148 (134–161)	148 (130–162)	0.700	1.004 (0.989–1.02)	0.578	1.014 (0.993–1.036)	0.184
HbA1c (%)	5.6 (5.4–6.2)	5.9 (5.5–6.6)	0.176	0.652 (0.343–1.238)	0.191	0.707 (0.368–1.358)	0.298
Cigarette	13 (40.6)	102 (35.9)	0.600	1.221 (0.579–2.574)	0.600	1.346 (0.413–4.379)	0.622

*Md, median; IOR, interquartile rage; N, number; cOR, crude odds ratio; aOR, adjusted odds ratio; AF, atrial fibrillation; SBP, systolic blood pressure.*

*Adjusting for AF, age, SBP, HbA1c, cigarette.

## Discussion

Contrary to the observations of increased hemorrhage rates and worse functional outcomes in patients with AF-associated stroke who underwent supportive care and/or IVT during the pre-endovascular era, when MT was not yet widely available (5–7), comorbid AF was associated with faster procedural time and increased rates of first-pass success without the increased risk of intracranial hemorrhage or worse functional outcomes for anterior circulation ischemic stroke treated with MT. However, whether these associations exist in posterior circulation ischemic stroke remains unclear.

Here, we found no significant difference in the safety and efficacy of MT for ABAO, regardless of whether patients had comorbid AF. Moreover, patients with AF had a higher rate of ischemic recurrence within 1 year after MT.

Despite a higher admission NIHSS score or pc-ASPECTS and a higher incidence of a maximum neurological deficit from the onset, there were not much worse functional outcomes for ABAO treated with MT. Surprisingly, patients with AF were more likely to have an intracranial hemorrhage, but we found that comorbid AF did not increase the rate of intracranial hemorrhage in patients undergoing MT, which is consistent with the results of the abovementioned studies on anterior circulation ischemic stroke. This is contrary to the common viewpoint in the pre-endovascular era that AF was a predictor of intracranial hemorrhage (25).

Specifically, in our study, the high odds of ischemic recurrence between the AF and non-AF cohorts were 17.6 and 8.1%, respectively (*p* = 0.020). In the univariate and multivariate analyses, AF status was significantly associated with recurrence. A previous study showed that medication for secondary prevention was insufficiently administered to eligible patients (26). Another study showed that anticoagulants could reduce the risks of ischemic stroke events in patients with ischemic stroke and AF (27). According to a review of stroke in China, only 30% of patients with ischemic stroke and AF received oral anticoagulants at discharge, and 10% received oral anticoagulants 1 year after stroke (2). Aside from the concern over bleeding risk among patients, the poor chronic disease management by both doctors and patients, inconvenient monitoring of vitamin K antagonists, ineffective warfarin dosing, and high costs might have also contributed to the unsatisfactory maintenance of medication for 1 year. This suggests that some developing countries or countries with a serious aging population should pay greater attention to the quality of AF management and secondary prevention of AF-associated stroke to prevent more serious clinical outcomes caused by recurrence.

The strengths of our study include leveraging real-world data from a large multicenter database with > 600 thrombectomies for ABAO, which is a rare intracerebral vascular disease. Moreover, to our knowledge, this is the first report on comorbid AF as a predictor of ischemic recurrence, and our findings stressed the importance of managing secondary prevention in relation to AF.

However, certain limitations should be considered. First, our study has all the inherent limitations of a non-randomized study. The reasons for clinicians to select a specific treatment option are more complex than can be met by the scope of a prospective observational study. Multivariable analyses can never adjust completely for systematic differences between AF and non-AF cohorts. Second, due to the rare occurrence of basilar artery occlusion, there was a large difference in the number of patients enrolled between the AF and non-AF cohorts. Nevertheless, there was an adequate number of cases to perform a statistical analysis and obtain a credible result. Third, due to the poor compliance of patients, we did not have available data on the anticoagulant strategies or detailed information on the dosages of anticoagulants after discharge. Therefore, we were not able to analyze ischemic recurrence simultaneously. Despite these limitations, our findings still constitute one of the best available data for ischemic stroke in patients with ABAO and comorbid AF.

## Conclusions

Our findings revealed no significant difference in the safety and efficacy of MT for ABAO, regardless of whether patients had comorbid AF. However, patients with AF had a higher rate of ischemic recurrence within 1 year after MT. Reducing the recurrence rate of stroke by providing ongoing secondary prevention measures may be the crucial strategy to improve long-term outcomes for patients with ABAO.

## Data availability statement

The original contributions presented in the study are included in the article/supplementary material, further inquiries can be directed to the corresponding authors.

## Ethics statement

The studies involving human participants were reviewed and approved by the local institutional review board (Medical Ethics Committee of Second Affiliated Hospital of Third Military Medical University, PLA Approve Number:201308701). The patients/participants provided their written informed consent to participate in this study. Written informed consent was obtained from the individual(s) for the publication of any potentially identifiable images or data included in this article.

## Author contributions

WZ was responsible for conceptualization, supervision, and writing—review & editing. QY was responsible for conceptualization, funding acquisition, supervision, and writing—review & editing. CZ, WL, and XL was responsible for investigation, data curation, formal analysis, and writing- original draft. JL, JS, SL, JcH, and WK were responsible for investigation and data curation. JrH, JjY, RS, CY, DX, LL, HS, FL, ZQ, and DW were responsible for the investigation. All authors contributed to the article and approved the submitted version.

## Funding

This work was supported by the National Natural Science Foundation of China (No.: 82071323), Chongqing Major Disease Prevention and Control Technology Research Project (No.: 2019ZX001), Chongqing Natural Science Foundation (No.: cstc2020jcyj-msxmX0926), Chongqing Science and Health Joint Project (No.: 2019ZDXM002), Clinical Medical Research Personnel Training Program (No.: 2018XLC1005), and Sichuan Medical Science and Technology Program (No.: 18PJ337).

## Conflict of interest

The authors declare that the research was conducted in the absence of any commercial or financial relationships that could be construed as a potential conflict of interest.

## Publisher's note

All claims expressed in this article are solely those of the authors and do not necessarily represent those of their affiliated organizations, or those of the publisher, the editors and the reviewers. Any product that may be evaluated in this article, or claim that may be made by its manufacturer, is not guaranteed or endorsed by the publisher.

## References

[1] Collaborators GBDS. Global, regional, and national burden of stroke and its risk factors, 1990-2019: a systematic analysis for the global burden of disease study 2019. Lancet Neurol. (2021) 20:795–820. 10.1016/S1474-4422(21)00252-0 34487721 PMC8443449

[2] Wu S Wu B Liu M Chen Z Wang W Anderson CS. Stroke in China: advances and challenges in epidemiology, prevention, and management. Lancet Neurol. (2019) 18:394–405. 10.1016/S1474-4422(18)30500-3 30878104

[3] Ornello R Degan D Tiseo C Di Carmine C Perciballi L Pistoia F. Distribution and temporal trends from 1993 to 2015 of ischemic stroke subtypes: a systematic review and meta-analysis. Stroke. (2018) 49:814–9. 10.1161/STROKEAHA.117.020031 29535272

[4] Li Z Jiang Y Li H Xian Y Wang Y. China's response to the rising stroke burden. BMJ. (2019) 364:l879. 10.1136/bmj.l879 30819725 PMC6394375

[5] Saposnik G Gladstone D Raptis R Zhou L Hart RG. Atrial fibrillation in ischemic stroke: predicting response to thrombolysis and clinical outcomes. Stroke. (2013) 44:99–104. 10.1161/str.44.suppl_1.ATMP75 23168456

[6] Seet RCS Zhang Y Wijdicks EF Rabinstein AA. Relationship between chronic atrial fibrillation and worse outcomes in stroke patients after intravenous thrombolysis. Arch Neurol. (2011) 68:1454–8. 10.1001/archneurol.2011.248 22084129

[7] Steger C Pratter A Martinek-Bregel M Avanzini M Valentin A Slany J. Stroke patients with atrial fibrillation have a worse prognosis than patients without: data from the Austrian stroke registry. Eur Heart J. (2004) 25:1734–40. 10.1016/j.ehj.2004.06.030 15451152

[8] Kimura K Minematsu K Yamaguchi T. Atrial fibrillation as a predictive factor for severe stroke and early death in 15,831 patients with acute ischaemic stroke. J Neurol Neurosurg Psychiatry. (2005) 76:679–83. 10.1136/jnnp.2004.048827 15834026 PMC1739612

[9] Giray S Ozdemir O Baş DF Inanç Y Arlier Z Kocaturk O. Does stroke etiology play a role in predicting outcome of acute stroke patients who underwent endovascular treatment with stent retrievers? J Neurol Sci. (2017) 372:104–9. 10.1016/j.jns.2016.11.006 28017193

[10] Akbik F Alawieh A Cawley CM Howard BM Tong FC Nahab F. Differential effect of mechanical thrombectomy and intravenous thrombolysis in atrial fibrillation associated stroke. J Neurointerv Surg. (2021) 13:883–8. 10.1136/neurintsurg-2020-016720 33318066 PMC8377613

[11] Mattle HP Arnold M Lindsberg PJ Schonewille WJ Schroth G. Basilar artery occlusion. Lancet Neurol. (2011) 10:1002–14. 10.1016/S1474-4422(11)70229-0 22014435

[12] Shuaib A Butcher K Mohammad AA Saqqur M Liebeskind DS. Collateral blood vessels in acute ischaemic stroke: a potential therapeutic target. Lancet Neurol. (2011) 10:909–21. 10.1016/S1474-4422(11)70195-8 21939900

[13] Seiffge DJ Traenka C Polymeris A Hert L Peters N Lyrer P. Early start of DOAC after ischemic stroke: risk of intracranial hemorrhage and recurrent events. Neurology. (2016) 87:1856–62. 10.1212/WNL.0000000000003283 27694266

[14] Writing Group for the BG Zi W Qiu Z Wu D Li F Liu H . Assessment of endovascular treatment for acute basilar artery occlusion *via* a nationwide prospective registry. JAMA Neurol. (2020) 77:561–73. 10.1001/jamaneurol.2020.0156 32080711 PMC7042866

[15] Hindricks G Potpara T Dagres N Arbelo E Bax JJ Blomström-Lundqvist C. 2020 ESC Guidelines for the diagnosis and management of atrial fibrillation developed in collaboration with the European Association for Cardio-Thoracic Surgery (EACTS): the task force for the diagnosis and management of atrial fibrillation of the European Society of Cardiology (ESC) developed with the special contribution of the European Heart Rhythm Association (EHRA) of the ESC. Eur Heart J. (2021) 42:373–498. 10.1093/eurheartj/ehab648 32860505

[16] Lip GYH Hunter TD Quiroz ME Ziegler PD Turakhia MP. Atrial fibrillation diagnosis timing, ambulatory ECG monitoring utilization, and risk of recurrent stroke. Circ Cardiovasc Qual Outcomes. (2017) 10:e002864. 10.1161/CIRCOUTCOMES.116.002864 28096204

[17] Brott T Adams HP Olinger CP Marler JR Barsan WG Biller J. Measurements of acute cerebral infarction: a clinical examination scale. Stroke. (1989) 20:864–70. 10.1161/01.STR.20.7.864 2749846

[18] Puetz V Sylaja PN Coutts SB Hill MD Dzialowski I Mueller P. Extent of hypoattenuation on CT angiography source images predicts functional outcome in patients with basilar artery occlusion. Stroke. (2008) 39:2485–90. 10.1161/STROKEAHA.107.511162 18617663

[19] Adams HP Jr Bendixen BH Kappelle LJ Biller J Love BB Gordon DL. Classification of subtype of acute ischemic stroke. Definitions for use in a multicenter clinical trial. TOAST. Trial of Org 10172 in Acute Stroke Treatment. Stroke. (1993) 24:35–41. 10.1161/01.STR.24.1.35 7678184

[20] Tomsick T Broderick J Carrozella J Khatri P Hill M Palesch Y. Revascularization results in the interventional management of stroke II trial. AJNR Am J Neuroradiol. (2008) 29:582–7. 10.3174/ajnr.A0843 18337393 PMC3056457

[21] Aubertin M Weisenburger-Lile D Gory B Richard S Blanc R Ducroux C. First-pass effect in basilar artery occlusions: insights from the endovascular treatment of ischemic stroke registry. Stroke. (2021) 52:3777–85. 10.1161/STROKEAHA.120.030237 34433309

[22] van Swieten JC Koudstaal PJ Visser MC Schouten HJ van Gijn J. Interobserver agreement for the assessment of handicap in stroke patients. Stroke. (1988) 19:604–7. 10.1161/01.STR.19.5.604 3363593

[23] von Kummer R Broderick JP Campbell BCV Demchuk A Goyal M Hill MD. The Heidelberg bleeding classification: classification of bleeding events after ischemic stroke and reperfusion therapy. Stroke. (2015) 46:2981–6. 10.1161/STROKEAHA.115.010049 26330447

[24] Turc G Lee J-Y Brochet E Kim JS Song J-K Mas J-L. Atrial septal aneurysm, shunt size, and recurrent stroke risk in patients with patent foramen ovale. J Am Coll Cardiol. (2020) 75:2312–20. 10.1016/j.jacc.2020.02.068 32381162

[25] Wahlgren N Ahmed N Eriksson N Aichner F Bluhmki E Davalos A. Multivariable analysis of outcome predictors and adjustment of main outcome results to baseline data profile in randomized controlled trials: Safe Implementation of Thrombolysis in Stroke-MOnitoring STudy (SITS-MOST). Stroke. (2008) 39:3316–22. 10.1161/STROKEAHA.107.510768 18927461

[26] Kim JM Park KY Yu IW Song TJ Kim YJ Kim BJ. Incidence of oral anticoagulant interruption among stroke patients with atrial fibrillation and subsequent stroke. Eur J Neurol. (2020) 27:900–2. 10.1111/ene.14175 32064742

[27] Aguilar MI Hart R. Oral anticoagulants for preventing stroke in patients with non-valvular atrial fibrillation and no previous history of stroke or transient ischemic attacks. Cochrane Database Syst Rev. (2005) 2005:CD001927. 10.1002/14651858.CD001925.pub2 16034869 PMC8408914

